# The *BRCA2* mutation status shapes the immune phenotype of prostate cancer

**DOI:** 10.1007/s00262-019-02393-x

**Published:** 2019-09-23

**Authors:** Maximilian Jenzer, Peter Keß, Cathleen Nientiedt, Volker Endris, Maximilian Kippenberger, Jonas Leichsenring, Fabian Stögbauer, Josh Haimes, Skyler Mishkin, Brian Kudlow, Adam Kaczorowski, Stefanie Zschäbitz, Anna-Lena Volckmar, Holger Sültmann, Dirk Jäger, Anette Duensing, Peter Schirmacher, Markus Hohenfellner, Carsten Grüllich, Albrecht Stenzinger, Stefan Duensing

**Affiliations:** 1 Section of Molecular Urooncology, Department of Urology, University of Heidelberg School of Medicine, Im Neuenheimer Feld 517, 69120 Heidelberg, Germany; 2 Department of Medical Oncology, University of Heidelberg School of Medicine, National Center for Tumor Diseases (NCT), Im Neuenheimer Feld 460, 69120 Heidelberg, Germany; 3 Institute of Pathology, University of Heidelberg School of Medicine, Im Neuenheimer Feld 224, 69120 Heidelberg, Germany; 4 ArcherDX, 2477 55th Street, Boulder, CO 80301 USA; 5 National Center for Tumor Diseases, German Cancer Research Center, Cancer Genome Research, Im Neuenheimer Feld 460, 69120 Heidelberg, Germany; 6 German Cancer Consortium (DKTK), Heidelberg, Germany; 7 Cancer Therapeutics Program, UPMC Hillman Cancer Center, Pittsburgh, USA; 8 Department of Pathology, University of Pittsburgh School of Medicine, 5117 Centre Avenue, Pittsburgh, PA 15213 USA; 9 Section of Precision Oncology of Urological Malignancies, Department of Urology, University of Heidelberg School of Medicine, Im Neuenheimer Feld 517, 69120 Heidelberg, Germany; 10 Department of Urology, University of Heidelberg School of Medicine, Im Neuenheimer Feld 110, 69120 Heidelberg, Germany; 11 Section of Translational Urooncology, Department of Medical Oncology, University of Heidelberg School of Medicine, National Center for Tumor Diseases (NCT), Im Neuenheimer Feld 460, 69120 Heidelberg, Germany

**Keywords:** Prostate cancer, Homologous recombination deficiency, *BRCA1/2*, Tumor-infiltrating lymphocytes, Tumor microenvironment, Immune checkpoint inhibitors

## Abstract

Defects in DNA damage repair caused by mutations in *BRCA1/2, ATM* or other genes have been shown to play an important role in the development and progression of prostate cancer. The influence of such mutations on anti-tumor immunity in prostate cancer, however, is largely unknown. To better understand the correlation between *BRCA1/2* mutations and the immune phenotype in prostate cancer, we characterized the immune infiltrate of eight *BRCA2*-mutated tumors in comparison with eight *BRCA1/2* wild-type patients by T-cell receptor sequencing and immunohistochemistry for CD45, CD4, CD8, FOXP3, and CD163. In addition, we analyzed seven prostate cancer biopsies that were either *BRCA2* or *ATM*-mutated in comparison with wild-type tumors. Whereas in *BRCA1/2* wild-type tumors, immune cells were found predominantly extratumorally, most *BRCA2*-mutated tumors including one biopsy showed a significantly increased intratumoral immune cell infiltration. The ratio of intratumoral to extratumoral immune cells was considerably higher in *BRCA2*-mutated tumors for all markers and reached statistical significance for CD4 (*p* = 0.007), CD8 (*p* = 0.006), and FOXP3 (*p* = 0.001). However, the intratumoral CD8 to FOXP3 ratio showed a trend to be lower in *BRCA2*-mutated tumors suggesting a more suppressed tumor immune microenvironment. Our findings provide a rationale for the future use of immune oncological approaches in *BRCA2*-mutated prostate cancer and may encourage efforts to target immunosuppressive T-cell populations to prime tumors for immunotherapy.

**Electronic supplementary material:**

The online version of this article (10.1007/s00262-019-02393-x) contains supplementary material, which is available to authorized users.

## Introduction

Prostate cancer is the most common non-cutaneous cancer in men and the second leading cause of death from cancer in men in the USA and worldwide [1, 2]. Defects in DNA damage repair are increasingly recognized to define a subgroup of prostate cancer patients with more aggressive tumor growth and earlier onset of disease [3]. *BRCA1* and *BRCA2* are two genes involved in homologous recombination (HR)-mediated repair of DNA double strand breaks and have first been described as tumor suppressor genes in hereditary breast cancer [4, 5]. HR deficiency caused by hereditary and/or somatic mutations in *BRCA1/2* or other DNA repair genes have been found not only in breast cancer but also in other types of cancer including ovarian or prostate cancer [3]. Mutations in *BRCA1/2* lead to an increase of the somatic mutation rate [6–8], indels, and chromosome copy number alterations (CNAs) [9] and, therefore, possibly promote the formation of neoepitopes [10]. *BRCA1/2*-deficient prostate cancers not only show increased sensitivity to platinum-based chemotherapies [11], but also to poly-(ADP-ribose) polymerase (PARP) inhibitors [12–14], thus giving *BRCA1/2* deficiency a high clinical relevance in these patients [15, 16].

Immune checkpoint inhibitors targeting CTLA-4 or PD-1/PD-L1 are increasingly used therapeutically in a number of tumor entities including renal cell carcinoma and bladder cancer [17]. Biomarkers predicting the response to immune checkpoint inhibitors include PD-L1 status [18], mutational load [7], and neoepitope formation [19] among others. Prostate cancer is known to have a relatively low mutational load in comparison with other epithelial malignancies [20, 21]. In line with this finding, the median progression-free survival (PFS) in a phase III trial using the anti-CTLA-4 antibody ipilimumab in asymptomatic or minimally symptomatic metastatic castration-resistant prostate cancer (mCRPC) patients was only 5.6 months in the treatment arm vs. 3.8 months in the placebo arm with no difference in the median overall survival (OS, 28.7 vs. 29.7 months) [22]. In another phase III trial, ipilimumab was compared to placebo in mCRPC patients who progressed after docetaxel and had received radiotherapy for bone metastases with no difference in the median OS (11.2 vs. 10.0 months) [23]. While a phase I study with the anti-PD-1 antibody nivolumab showed no response in 13 mCRPC patients [24], preliminary data from a phase II trial of the anti-PD-1 antibody pembrolizumab in 258 patients with docetaxel-refractory mCRPC showed a 5% objective response rate (ORR) regardless of PD-L1 status, but an ORR of 12% in patients with somatic *BRCA1/2* or *ATM* mutations [25]. The latter finding is in line with results, showing that *BRCA2* mutations are enriched in melanoma patients responding to anti-PD-1 therapy [26]. Since HR deficiency causes increased mutational load thereby potentially creating neoepitopes, it could be used to define a subgroup of prostate cancer patients who would potentially benefit from immune checkpoint inhibitors.

A number of studies have reported that the infiltration with CD4- or CD8-positive lymphocytes is increased in prostate cancer compared to benign prostate tissue [27]. However, other reports did not find such an association [28]. T-cell infiltration has been reported to increase with androgen deprivation [29, 30], but almost nothing is known about the immune milieu in mCRPC [31]. In contrast to most other cancers, a high number of CD8-positive TILs in prostate cancer appear to be associated with a poor prognosis including a shorter time to biochemical and clinical progression, castration resistance, and/or metastatic dissemination [32, 33]. Similar results have been reported for a high numbers of CD4-positive TILs [34, 35]. The reason for this observation may be a dysfunction or a suppression of T cells, e.g., through PD-L1 [33] or CD73 [36]. In addition, immunosuppressive cells such as FOXP3-positive regulatory T cells and CD163-positive tumor-associated (M2) macrophages were also found to be enriched in prostate cancer and associated with a more unfavorable patient outcome [28, 37].

It has previously been shown in breast cancer that HR deficiency caused by *BRCA1* inactivation is accompanied by an increase of tumor infiltration with CD4-positive T cells, CD8-positive cytotoxic T cells, PD-L1 expression, and possibly response to immune checkpoint inhibition [10, 38, 39]. Remarkably, a higher abundance of TILs was not found in *BRCA2*-mutated breast cancer, despite similar genomic alterations, suggesting that additional mechanisms than the mutational burden may shape cellular immune responses [39]. A relative deficiency in TILs was also detected in *BRCA2*-mutated ovarian cancer when compared to *BRCA1*-mutated tumors [40]. However, other studies found increased numbers of CD3- and CD8-positive TILs in *BRCA1/2*-mutated ovarian cancer without significant difference between *BRCA1* or *BRCA2* mutations [10]. The impact of *BRCA1/2* mutations on the cellular immune phenotype of prostate cancer is largely unknown.

To better understand the impact of *BRCA1/2* mutations on the immune phenotype of prostate cancer, we initiated a proof-of-concept study to characterize the cellular immune infiltrate of eight *BRCA2* mutated in comparison with eight *BRCA1/2* wild-type prostate cancer patients by T-cell receptor (TCR)-sequencing and immunohistochemistry (IHC) for CD45, CD4, CD8, FOXP3, and CD163. In addition, we characterized the immune infiltrate in seven prostate cancer biopsies that were either *BRCA2* or *ATM* mutated or wild type. Results show an increased number of tumor-infiltrating lymphocytes, including potentially immunosuppressive FOXP3-positive lymphocytes, in *BRCA2*-mutated prostate cancers compared to the *BRCA1/2* wild-type group, which harbored mostly extratumoral immune cells. Our findings provide a rationale for the future use of immune oncological agents in *BRCA2*-mutated prostate cancer patients and may encourage efforts to target immunosuppressive T-cell populations.

## Methods

### Patient samples and targeted next-generation sequencing (NGS)

In this retrospective proof-of-concept study, formalin-fixed, paraffin-embedded (FFPE) tissue sections from 16 men with prostate adenocarcinoma and known *BRCA1* and *BRCA2* mutation status were analyzed. Eight patients were *BRCA2* mutated, and one patient had a confirmed germline mutation. The other eight patients were a control group matched for age, Gleason Score, initial PSA, and initial TNM-state, but were *BRCA2* wild type. All 16 patients were *BRCA1* wild type. The mutation status of *BRCA1* and *BRCA2* was determined by targeted next generations sequencing of tumor tissue as previously reported [16]. In addition, FFPE prostate biopsy samples from seven patients were analyzed after mutation testing by targeted NGS using a panel of 37 DNA damage repair and checkpoint genes (*ATM, ATR, BARD1, BRCA1, BRCA2, BRIP1, CHEK1, CHEK2, FAM175A, FANCA, FANCB, FANCC, FANCD2, FANCE, FANCF, FANCG, FANCI, FANCL, FANCM, ERCC2, ERCC4, ERCC5, MLH1, MSH2, MSH6, PALB2, PMS1, PMS2, RAD50, RAD51C, RAD51D, RECQL4, MRE11A, NBN, SLX4, TP53, XRCC2).* Amplicon library preparation was performed with the Ion AmpliSeq Library Kit v2.0 (Thermo Fisher Scientific, Waltham, MA, USA) [41]. After the PCR reaction, primer end sequences were partially digested using FuPa reagent, followed by the ligation of barcoded sequencing adapters (Ion Xpress Barcode Adapters, Life Technologies). The final libraries were diluted to a concentration of 50 pM and processed on Ion Chef (Thermo Fisher Scientific). Sequencing was performed on an Ion S5XL/Prime sequenzer using 520/530 chips with a mean coverage per amplicon between 1000 and 3000 fold. Data analysis, variant calling, and annotation were performed as previously described [42–44]. All patients included in the analysis were of Caucasian descent.

### T-cell receptor (TCR)-sequencing

RNA from the aforementioned FFPE tissue samples was extracted with the Maxwell RSC RNA FFPE Kit (Promega, Madison, WI, USA) according to the manufacturer’s protocol with the exception that the DNase step was omitted. RNA was quantified with Qubit RNA HS Assay Kit (Thermo Fisher Scientific, Waltham, MA, USA) after purification. Between 786 to 1000 ng of RNA was used as input for library generation with the Archer Immunoverse™-HS TCR beta/gamma Kit, for Illumina (DB0232; ArcherDX, Boulder, CO, USA) following protocol revision LA092.A. All purifications during library preparation were performed with Agencourt AMPure XP (Beckman Coulter, Brea, CA, USA). Final libraries were quantified using the KAPA Library Quantification Kit (KAPA Biosystems, Wilmington, MA, USA) and pooled to equimolar concentration. The intended concentration of pooled libraries was confirmed with the KAPA Library Quantification Kit.

Libraries were sequenced on an Illumina NextSeq 500 using NextSeq 500 v2 reagents (Illumina, San Diego, CA, USA) for paired end, 150 base pair reads, and dual index reads. Libraries were multiplexed, such that an average of 1.86 million paired reads was attributed to each library. The flow cell was loaded with 1.6 pM denatured library and 20% PhiX Control v3 (Illumina).

Data were analyzed by Archer Analysis version 5.1.3 (ArcherDX). Briefly, adapter sequences are trimmed from the reads, and then, PCR duplicates are collapsed using molecular barcodes to identify unique molecules. Consensus reads representing unique input molecules are passed to MiXCR for V-(D)-J segment mapping and clonotype assembly. When used alone, “clones” means the number of total identified clones, while “clonotypes” means the number of identified unique sequences.

### Immunohistochemistry

Consecutive FFPE tissue sections were deparaffinized in xylene and rehydrated in a graded ethanol series. Antigen retrieval was performed with a steam cooker using retrieval buffer (Dako, Agilent, Santa Clara, CA, USA,). Primary antibodies used were directed against Ki-67 (MIB-1, Dako, 1:100), CD4 (4B12, NCL-L-CD4-368, Leica, Wetzlar, Germany, 1:50), CD8 (144B, ab17147, Abcam, Cambridge, UK, 1:25), CD45 (2B11 + PD7/26, Dako, 1:100), CD163 (10D6, NCL-L-CD163, Leica, 1:100), FOXP3 (236A/E7, ab20034, Abcam, 1:100), and PD-L1 (SP142, ab228472, Abcam, 1:100) and were incubated overnight at 4 °C. All antibodies have previously been extensively validated [45–51]. Immunodetection was done with a biotinylated secondary goat anti-mouse IgG (H + L) antibody (31800, Invitrogen, Thermo Fisher Scientific, 1:200), streptavidin conjugated to horseradish peroxidase (Thermo Fisher Scientific) and 3,3′-diaminobenzidine chromogen (Thermo Fisher Scientific). Nuclear counterstaining was done with hematoxylin (Thermo Fisher Scientific).

For Ki-67, five representative 1430 × 1070 µm areas with more than 50% tumor cell content were photographed using a Leica DM5000 B microscope and the percentage of positive tumor cells was counted. For CD45, CD4, CD8, FOXP3, and CD163, three regions were defined: Intratumoral (IT), extratumoral with direct contact to the tumor (ET1, < 10 µm distance from tumor), and extratumoral with one high-power field (HPF, 40 × objective) distance (ET2, 500 µm distance from tumor) to control for non-tumor-associated intraprostatic chronic inflammation. The microscopic fields for our analyses were chosen to represent TIL heterogeneity. For example, if 20% of the IT area showed a high density of TILs and the other 80% a low density, we analyzed five fields total of which one was representative of the high-density area and four of the low-density area. Positive cells in five HPFs were counted for each patient and each region. All cell counts were performed in a blinded fashion and the tumor mutation status was unblinded only after all counts were completed. One main observer performed the counts (P. Keß) and two independent observers (M. Jenzer and S. Duensing) performed additional counts on selected cases. Areas of acute inflammation and necrosis were excluded from the analyses.

### Statistical analysis

For statistical analyses, Student’s *t* test for independent samples, Mann–Whitney *U* test, or Fisher’s exact test was used wherever applicable. Differences with a *p* value of ≤ 0.05 were considered statistically significant. All data are available from the corresponding author at reasonable request.

## Results

### Enhanced intratumoral lymphocyte infiltration in *BRCA2*-mutated prostate cancers

Eight *BRCA2*-mutated prostate cancer patients and eight *BRCA1/2* wild-type patients matched for age, Gleason score, initial PSA, and initial TNM state were selected for this retrospective analysis. All patients and mutations have previously been reported in an unrelated study (Supplementary Table 1) [16]. *BRCA2*-mutated patients showed a trend towards a shorter progression-free survival (PFS; 22 vs. 34.9 months, *p* = 0.32) under androgen deprivation therapy and to shorter overall survival (OS, 43.3 vs. 58.5 months, *p* = 0.28), but differences in these and all other clinico-pathological characteristics were not statistically significant (Supplementary Table 2).

**Fig. 1:**
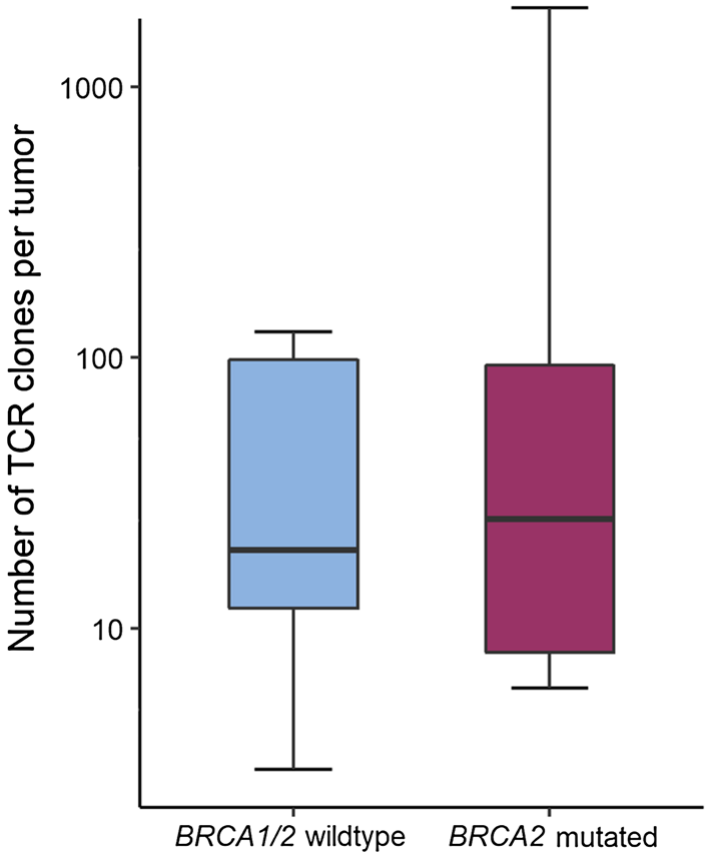
Number of TCR clones per tumor by *BRCA1/2* mutation status. Box plots represent the median number of identified TCR clones per sample in *BRCA1/2* wild-type (blue) and *BRCA2*-mutated (red) prostate cancer identified by TCR sequencing. Whiskers representing minimum and maximum of each group. A log_10_ scale is used for the *y*-axis

We first sought to explore the diversity of the T-cell infiltrate and performed T-cell receptor sequencing of the 16 tumors. Results showed a wide range in the number of T-cell clones in both *BRCA1/2* wild-type (median 20, range 3–124) and *BRCA2*-mutated (median 25.5, range 6–1954) tumors (Fig. 1). While the two tumors with the highest number of both total clones and unique clonotypes, respectively, were *BRCA2* mutated, there was overall no significant difference between *BRCA2*-mutated and *BRCA1/2* wild-type tumors (*p* = 1.0). The clonality as a diversity measure normalized to the number of unique T-cell clones, ranging from 0 (absolute polyclonal) to 1 (absolute monoclonal), was also similar (*p* = 0.26) between *BRCA1/2* wild-type (0.018) and *BRCA2*-mutated (0.032) tumors.

**Fig. 2:**
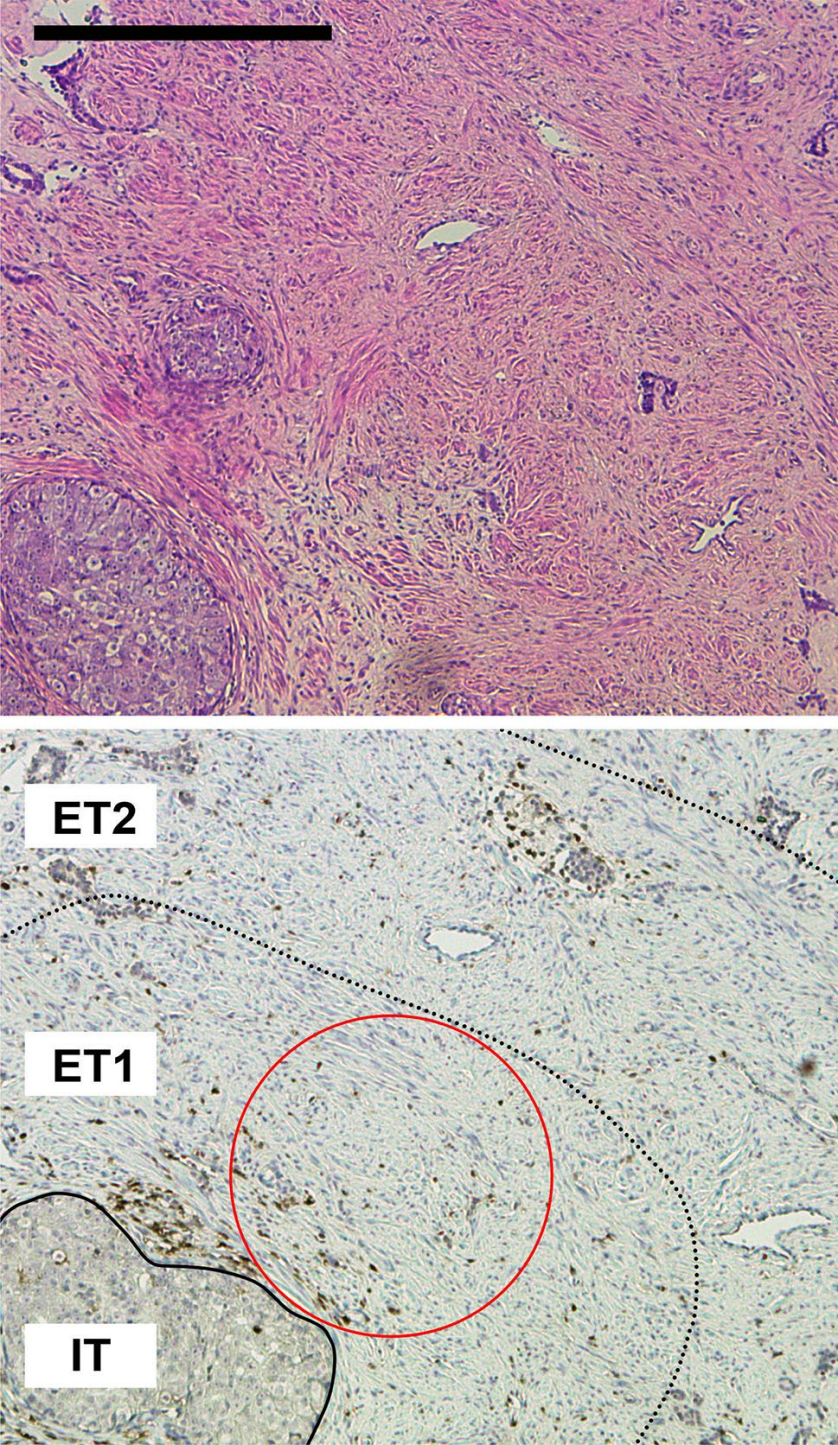
Overview of tumor and microenvironmental compartments analyzed. Representative H&E (top panel) and immunohistochemical (bottom panel) staining of prostate cancer for CD45 showing the three compartments intratumoral (IT), extratumoral 1 (ET1) and extratumoral 2 (ET2) used in this study. The red circle stands for a representative high-power field (40-fold objective, 500 µm in diameter) in the ET1 compartment. The scale bar represents 500 µm

**Fig. 3:**
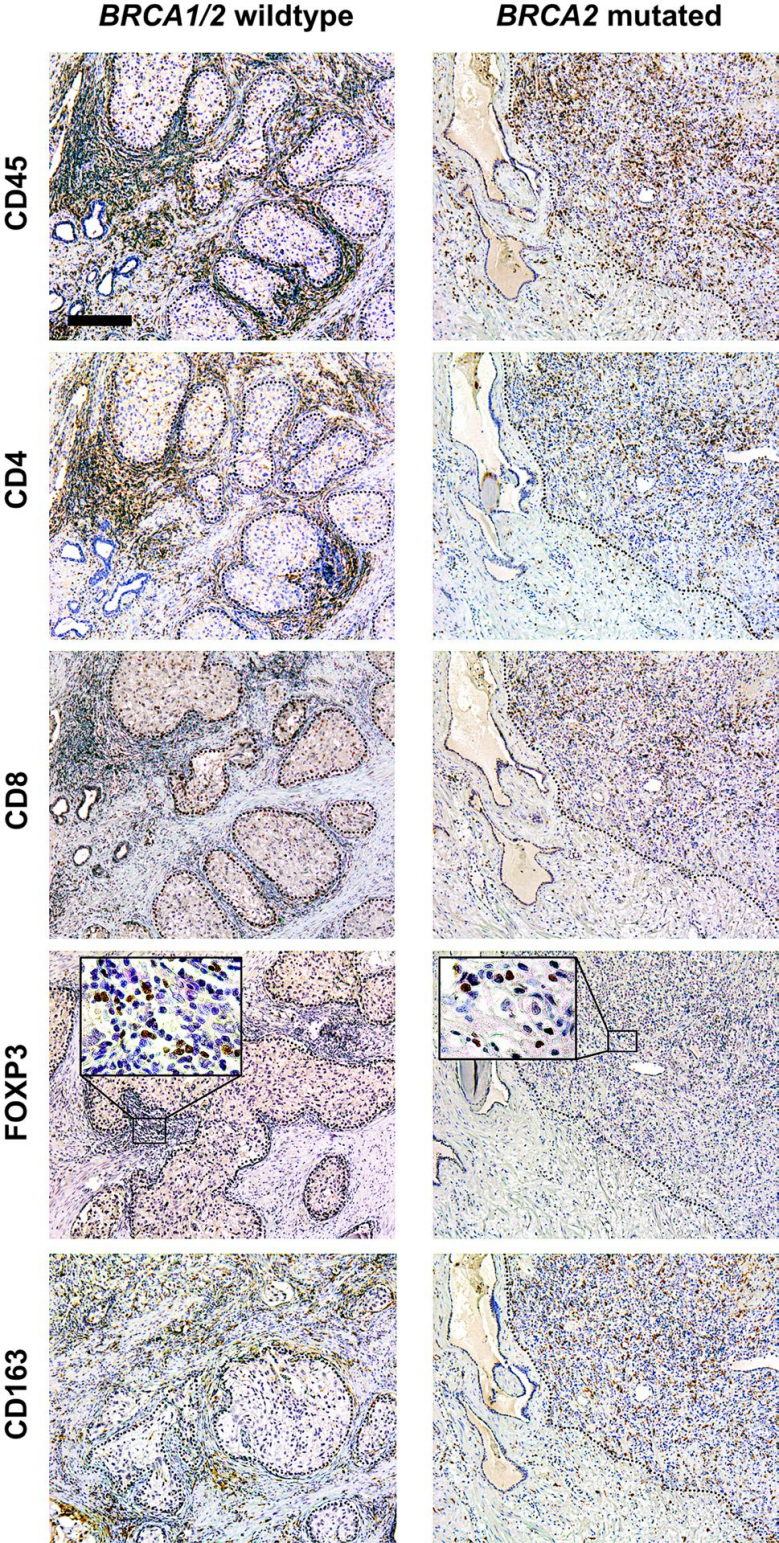
Overview of immunohistochemical stainings. Representative immunohistochemical stainings for CD45 (pan-leucocyte), CD4 (T helper cells), CD8 (cytotoxic T cells), FOXP3 (regulatory T cells), and CD163 (macrophages) on consecutive slides of a *BRCA1/2* wild-type and a *BRCA2*-mutated prostate cancer. The dotted line represents the border between tumor and stroma. Scale bar represents 200 µm

Based on these results, we decided to perform a detailed in situ analysis of the immune cell composition in different compartments of the tumors. An immunostochemical analysis for CD45, CD4, CD8, FOXP3, and CD163 was performed (Figs. 2, 3). In addition, tumors were stained for Ki-67 as a marker for cell proliferation. Altogether, a total of 1280 HPFs were analyzed in the 16 patients and in three distinct compartments: intratumoral (IT), extratumoral 1 (ET1), and extratumoral 2 (ET2), as illustrated in Fig. 2. Representative immunostainings in a *BRCA2*-mutated and a *BRCA1/2* wild-type tumor, respectively, are shown in Fig. 3.

**Fig. 4:**
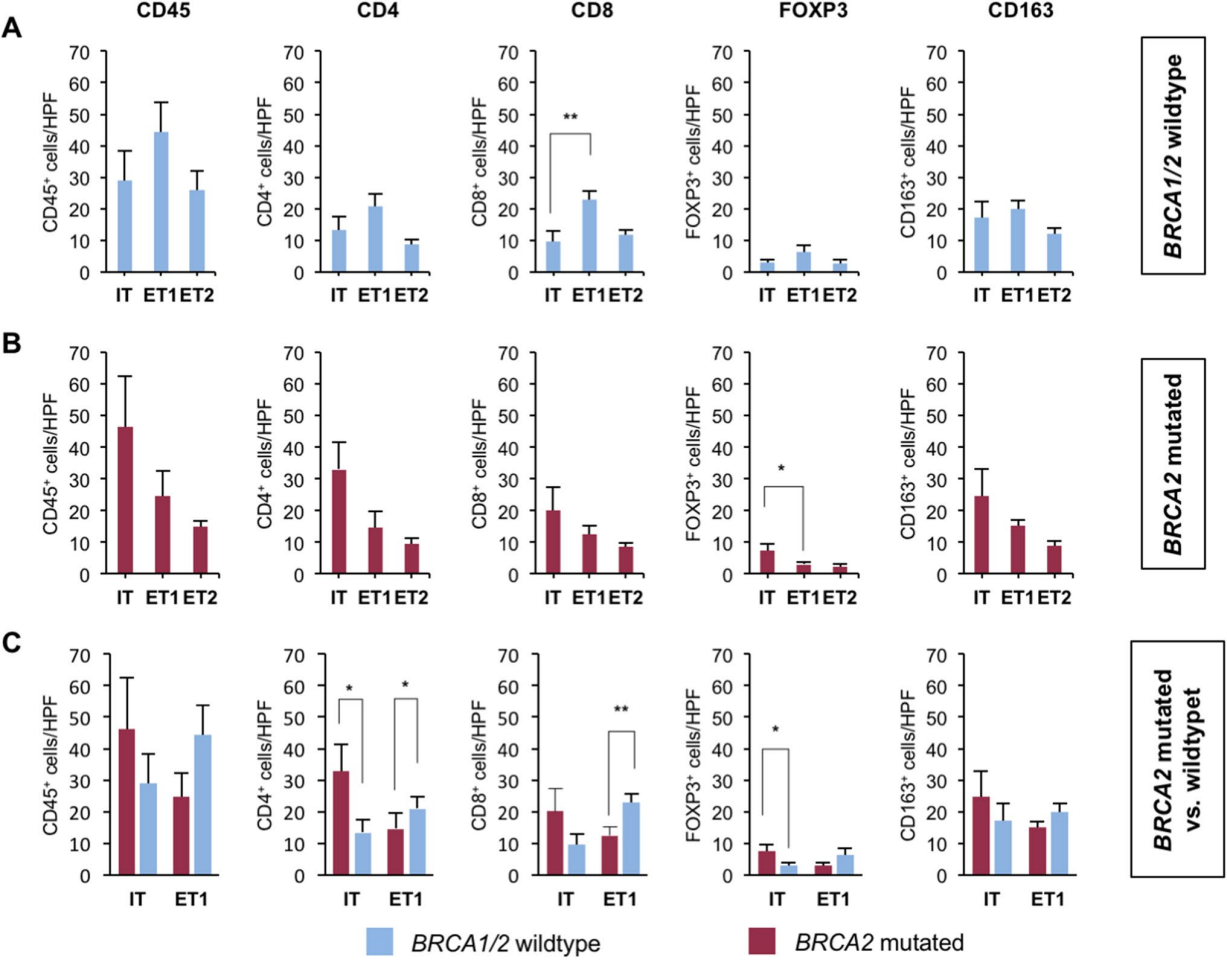
*BRCA2*-mutated tumors harbor more intratumoral immune cells. **a**, **b** Bar graphs show the mean number of positive cells (+ standard error) per 40 × HPF in *BRCA2*-mutated or *BRCA1/2* wild-type prostate cancer in the intratumoral (IT), extratumoral 1 (ET1), and extratumoral 2 (ET2) compartments for CD45, CD4, CD8, FOXP3, and CD163. **c** Bar graphs show the mean number of positive cells (+ standard error) per 40 × HPF in BRCA2-mutated compared to BRCA1/2 wild-type prostate cancer in the intratumoral (IT) and extratumoral 1 (ET1) compartments for CD45, CD4, CD8, FOXP3, and CD163. Each bar represents the mean of five 40 × HPF from the eight patients of each group i.e., *BRCA1/2* wild type and *BRCA2* mutated. BRCA1/2 wild-type tumors in blue, BRCA2-mutated tumors in red. Standard errors are shown. *p ≤ 0.05, **p ≤ 0.005

In *BRCA1/2* wild-type tumors (Fig. 4a), significantly more CD8 positive cells (mean 23 vs. 9.7 per HPF, *p* = 0.001) were detected in the ET1 compartment when compared to the IT compartment. There were also more CD4 positive cells (mean 21 vs. 13.4 per HPF, *p* = 0.10), FOXP3 positive cells (mean 6.2 vs. 2.8 per HPF, *p* = 0.10), CD45 positive cells (mean 44.3 vs. 29.1 per HPF, *p* = 0.17), and CD163 positive cells (mean 19.8 vs. 17.2 per HPF, *p* = 0.59) in the ET1 compartment than in the IT compartment, but differences did not reach statistical significance (Fig. 4a).

*BRCA2*-mutated tumors (Fig. 4b) harbored significantly more FOXP3 positive cells (mean 7.4 vs. 3.0 per HPF, *p* = 0.04) intratumorally than in the ET1 compartment. A similar trend towards an enrichment of immune cells in the IT compartment was found for CD4 positive cells (mean 32.9 vs. 14.6 per HPF, *p* = 0.07), CD45 positive cells (mean 46.3 vs. 24.6 per HPF, *p* = 0.16), CD163 positive cells (mean 24.6 vs. 15.1 per HPF, *p* = 0.2), and CD8 positive cells (mean 20.1 vs. 12.4 per HPF, *p* = 0.24) without reaching statistical significance (Fig. 4b).

We next compared the immune cell infiltration in the IT and ET1 compartments of both groups (Fig. 4c) and found that *BRCA2*-mutated tumors harbored significantly more CD4 positive cells (mean 32.9 vs. 13.4 per HPF, *p* = 0.02) and FOXP3 positive cells (mean 7.4 vs. 2.8 per HPF, *p* = 0.03) than *BRCA1/2* wild-type tumors. *BRCA2*-mutated tumors also showed a trend for more CD8 positive cells (mean 20.1 vs. 9.7 per HPF, *p* = 0.12) that did not reach statistical significance. The number of CD45 positive cells (mean 46.3 vs. 29.1 per HPF, *p* = 0.27) and CD163 positive cells (mean 24.6 vs. 17.2 per HPF, *p* = 0.36) was also higher but differences were, again, not statistically significant (Fig. 4c).

In the ET1 compartment, *BRCA1/2* wild-type tumors had significantly more CD8 positive cells (mean 23 vs. 12.4 per HPF, *p* = 0.004) and CD4 positive cells (mean 21 vs. 14.6 per HPF, *p* = 0.04) than *BRCA2*-mutated tumors. Furthermore, *BRCA1/2* wild-type tumors showed a trend towards more CD45 positive cells (mean 44.3 vs. 24.6 per HPF, *p* = 0.06), more CD163 positive cells (mean 19.8 vs. 15.3 per HPF, *p* = 0.10) and more FOXP3 positive cells (mean 6.2 vs. 3.0 per HPF, *p* = 0.11) in the ET1 compartment (Fig. 4c).

In the more distant ET2 compartment, *BRCA1/2* wild-type tumors showed a trend towards more CD45 positive cells (mean 25.9 vs. 14.9 per HPF, *p* = 0.05) and CD8 positive cells (mean 11.6 vs. 8.4 per HPF, *p* = 0.07) compared to *BRCA2*-mutated tumors. Differences for cells positive for CD163 (mean 11.9 vs. 8.9 per HPF, *p* = 0.38), FOXP3 (mean 2.7 vs. 2.2 per HPF, *p* = 0.56) and CD4 (mean 9.5 vs. 8.7 per HPF, *p* = 0.64) were not statistically significant (not shown).

*BRCA2*-mutated and *BRCA1/2* wild-type tumors showed no differences in the proliferation rate as determined by the percentage of Ki-67 positive tumor cells (mean 16.9 vs. 12.7% per HPF, *p* = 0.88, Supplementary Figure 1).

In addition, we analyzed the presence and frequency of PD-L1 positive TILs in *BRCA2*-mutated and wild-type patients. The overall frequency of these cells was low and slightly higher in *BRCA2*-mutated tumors (mean = 0.6 PD-L1 positive TILs per 40 × HPF) in comparison with wild-type tumors (mean = 0.38 per HPF) albeit without reaching statistical significance (*p* = 0.2).

Taken together, these results demonstrate that *BRCA2*-mutated tumors contain an increased number of intratumoral immune cells when compared to *BRCA1/2* wild-type tumors in particular CD4- and FOXP3-positive cells.

### Higher IT/ET1 ratio in *BRCA2*-mutated prostate cancers

**Fig. 5:**
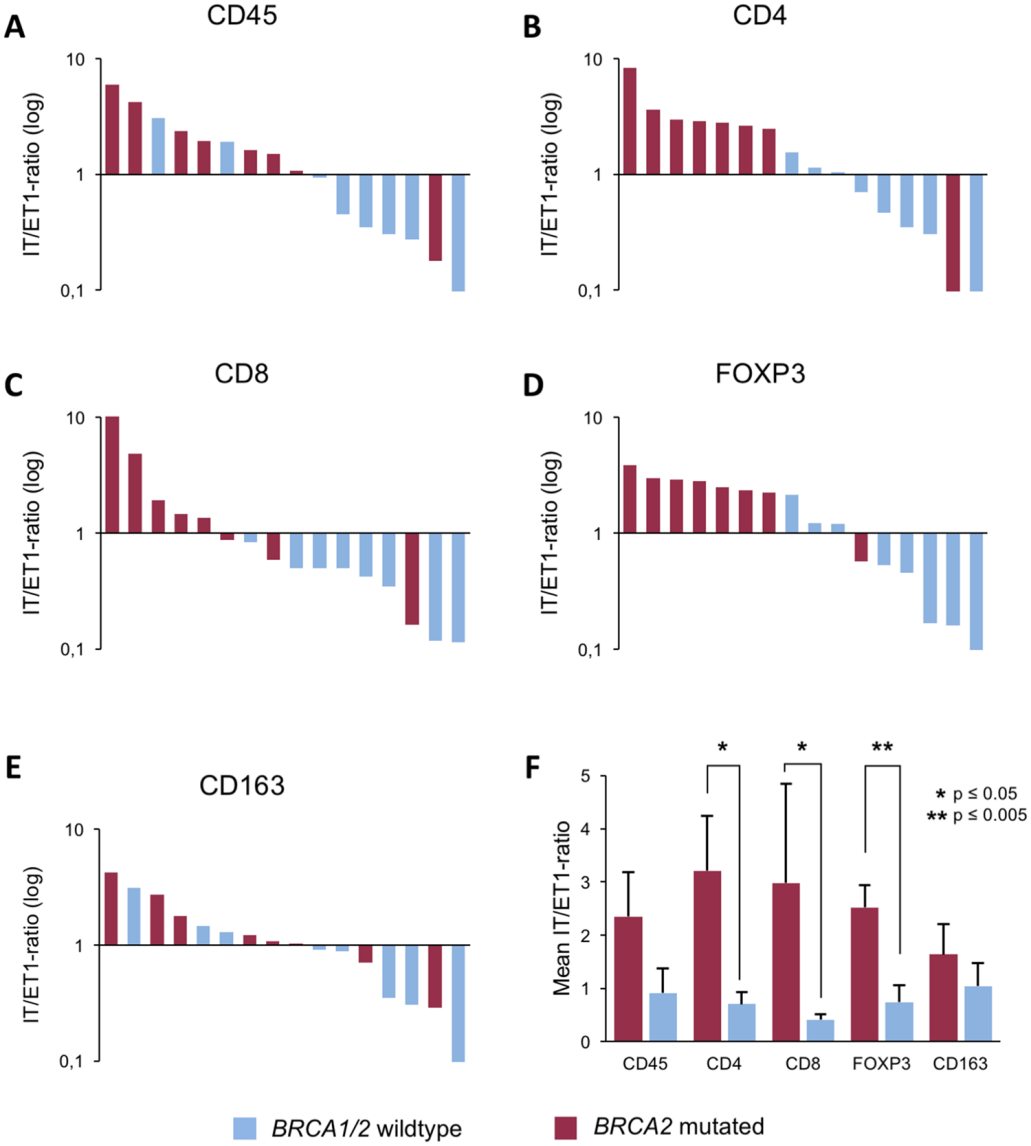
Shift in immune cell distribution in *BRCA2*-mutated tumors as reflected by the IT/ET ratio. Bar graphs show the ratio of mean positive cell counts from five representative HPF each of the intratumoral (IT) and extratumoral closest to the tumor (ET1) compartment of each patient sorted from highest to lowest for CD45 (**a**), CD4 (**b**), CD8 (**c**), FOXP3 (**d**), and CD163 (**e**). A log_10_ scale is used for the *y*-axis. **f** Bar graph showing mean IT/ET1 ratio for all eight patients of each group for CD45, CD4, CD8, FOXP3, and CD163. *BRCA1/2* wild-type tumors in blue, *BRCA2*-mutated tumors in red. Standard errors are shown. *p ≤ 0.05, **p ≤ 0.005

To further corroborate the notion that *BRCA2*-mutated tumors contain more intratumoral immune cells than *BRCA1/2* wild-type tumors, we calculated the ratio of intratumoral (IT) to directly extratumoral (ET1) positive cells for each staining and each patient (Fig. 5a–e). The mean IT/ET1 ratios were found to be significantly higher in *BRCA2*-mutated tumors than *BRCA1/2* wild-type tumors for CD4 (3.21 vs. 0.70, *p* = 0.007), CD8 (2.98 vs. 0.42, *p* = 0.006) and FOXP3 (2.52 vs. 0.74, *p* = 0.001) positive cells (Fig. 5f). For CD45 positive cells, the IT/ET1 ratio showed a trend to be higher in *BRCA2*-mutated tumors (2.95 vs. 0.92, *p* = 0.08) without reaching statistical significance, while for CD163 positive cells, the difference was not statistically significant (1.64 vs. 1.05, *p* = 0.38; Fig. 5f).

These results underscore the differences in the distribution of immune cells between *BRCA2*-mutated and *BRCA1/2* wild-type tumors in particular for the CD4-, CD8-, and FOXP3-positive lymphocytes.

### Lower intratumoral CD8/FOXP3 ratio in *BRCA2*-mutated tumors

**Fig. 6:**
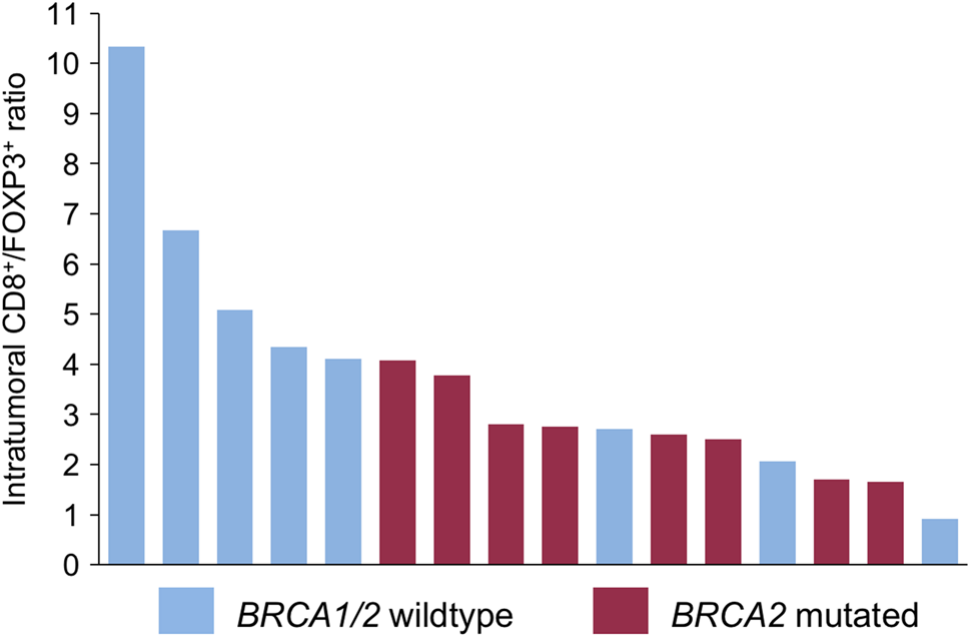
Intratumoral CD8/FOXP3 ratio is reduced in *BRCA2*-mutated tumors. Waterfall plot of intratumoral CD8/FOXP3 ratios in *BRCA1/2* wild-type (blue) and *BRCA2*-mutated (red) prostate cancer

Since not only cytotoxic CD8 positive T lymphocytes but also potentially immunosuppressive FOXP3 positive regulatory T cells were found to be increased intratumorally in *BRCA2*-mutated compared to *BRCA1/2* wild-type tumors, we calculated the CD8/FOXP3 ratio for each patient and each group. *BRCA2*-mutated tumors showed a trend towards lower CD8/FOXP3 ratios (Fig. 6), suggesting that they have more FOXP3-positive T cells in comparison with CD8 positive T cells, although the mean ratios of both groups were not significantly different (2.73 vs. 4.53, *p* = 0.12).

Taken together, these findings suggest differences not only in the IT and ET distribution of immune cells in *BRCA2*-mutated and wild-type prostate cancers, but also in the composition of TIL populations.

### Immune phenotype in prostate cancer biopsies

**Fig. 7:**
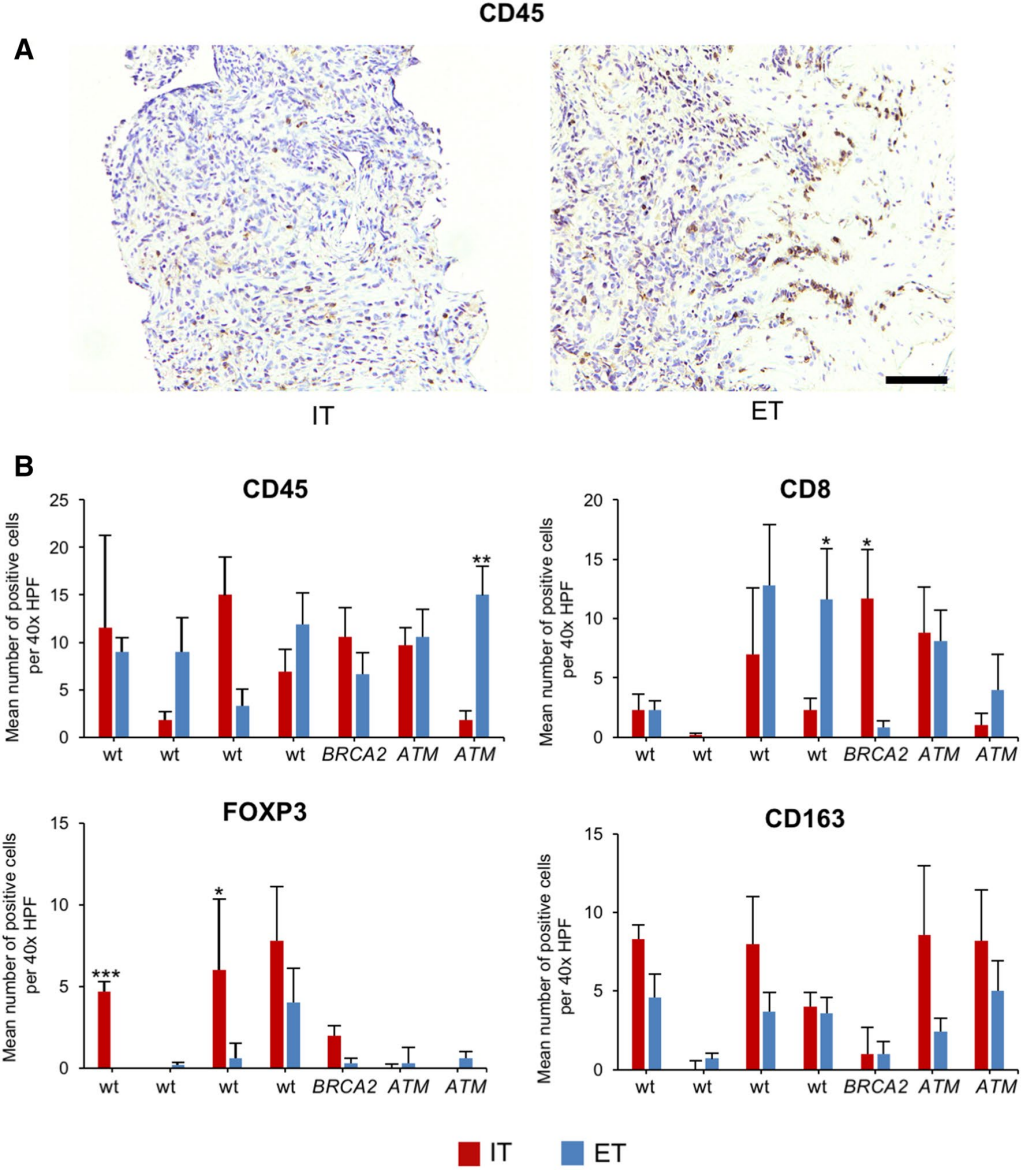
Immune phenotype of prostate cancer biopsies from *BRCA2-* or *ATM*-mutated tumors. **a** Representative immunohistochemical staining of prostate cancer biopsies for CD45 showing the intratumoral (IT) area (left) and an IT area with adjacent extratumoral (ET) area (right). The scale bar represents 250 µm. **b** Bar graphs show the mean number of positive cells per 40 × HPF in *BRCA2*-mutated or *ATM*-mutated biopsies compared to wild-type (wt) specimens in the intratumoral (IT) and extratumoral (ET) compartments for CD45, CD8, FOXP3, and CD163. Each bar represents counts from a mean of six 40 × HPFs. Standard errors are shown. *p ≤ 0.05, **p ≤ 0.005, ***p ≤ 0.0005

To confirm and extend our results, we next analyzed seven patients from which prostate cancer biopsies had been obtained and sequenced using a 37 gene panel (Fig. 7). One patient had a deleterious mutations in *BRCA2* (p.Glu2981fs*7, allele frequency 57.9%), two patients had a deleterious mutation in *ATM* (p.Arg2993*, allele frequency 40.2%; and p.Ser470*, allele frequency 40.4%), and four patients were wild-type for *BRCA2* and *ATM* and did not show any deleterious point mutations in any of the other 37 genes tested.

Because of the small size of prostate biopsies, analyses were limited to two areas, i.e., the intratumoral (IT) and extratumoral (ET) compartment after FFPE sections were stained for CD45, CD8, FOXP3, and CD163. The presence of a deleterious *BRCA2* mutation was found to correlate with a significantly higher number of intratumoral CD8 positive T lymphocytes (14.6-fold, *p* = 0.01, Fig. 7b). Remarkably, tumors with a deleterious mutation in *ATM* did not show more CD8 positive lymphocytes in the IT area, but more CD163 positive macrophages instead without reaching statistical significance. One *ATM*-mutated prostate cancer had a significantly higher frequency of extratumoral CD45 positive lymphocytes. The frequency of FOXP3-positive lymphocytes was overall low in tumors with a *BRCA2* or *ATM* mutation.

These results underscore that mutations in *BRCA2* shape the immune microenvironment, which can be detected even in prostate cancer biopsies.

## Discussion

The primary goal of this proof-of-concept study was to better understand the impact of the *BRCA1/2* mutation status on the immune phenotype of prostate cancer.

Our results show that *BRCA2*-mutated tumors harbor an enhanced intratumoral immune infiltrate compared to *BRCA1/2* wild-type tumors, in particular with respect to T lymphocytes expressing CD4, CD8, and FOXP3. Of note, there was a trend towards a lower intratumoral CD8^+^ to FOXP3^+^ ratio in *BRCA2*-mutated tumors that needs to be carefully interpreted, but suggests a more suppressed tumor immune microenvironment.

*BRCA2* mutations have been found to be associated with an increased mutational load [6–8], neoepitope formation [10], increased tumor-infiltrating lymphocytes [10], and a favorable response to immune checkpoint blockade [7, 25, 26]. However, the use of immune checkpoint inhibitors in advanced prostate cancer has been largely unsuccessful with respect to an improvement of overall patient survival thus far. Although a positive response to this treatment modality occurs frequently in patients with high intratumoral CD4- and CD8-positive T lymphocytes (“inflamed” tumors), their presence is clearly not sufficient for a response. The latter is also modulated by cells associated with immune suppression/immune homeostasis such as FOXP3-positive regulatory T cells, CD163-positive tumor-associated (M2) macrophages, or myeloid-derived suppressor cells (MDSCs). Hence, the presence of intratumoral T lymphocytes is necessary but clearly not sufficient for a response to immune checkpoint inhibitors [52], which makes treatment responses in *BRCA2*-mutated prostate cancer difficult to predict. Moreover, it is critical that cytotoxic T lymphocytes are capable of killing tumor cells, i.e., are not exhausted or dysfunctional. Recent approaches to target regulatory T cells may hence represent promising strategies to prime prostate cancer for immune checkpoint blockade [53, 54].

A subgroup analysis in the KEYNOTE-199 trial showed an increased, but still moderate ORR of 12% in *BRCA1/2*-mutated mCRPC [25] in response to pembrolizumab. DNA-damaging agents such as platinum salts or PARP inhibitors [55] may also increase the mutational load, the number of neoepitopes [56], and, therefore, possibly the response rate to immune checkpoint inhibitors and other immunotherapies. Results from ongoing studies focusing on combinations of PD1/PD-L1 antibodies with PARP inhibitors including nivolumab/rucaparib (CheckMate 9KD, NCT03338790), pembrolizumab/olaparib (KEYNOTE-365, NCT02861573), and durvalumab/olaparib (NCT02484404) [57] are, therefore, urgently awaited.

The reason for the relatively small difference in the number of TCR clones between *BRCA2*-mutated and wild-type tumors found in this study in comparison with the staining results is unclear. We attribute this result primarily to methodological differences, since no microdissection of tumor and stroma was performed prior to TCR sequencing. While other factors such as RNA degradation cannot be excluded, our finding showing the pronounced differences between intra- and extratumoral compartments in terms of lymphocyte infiltration dependent on the genetic background strongly argues for a tumor cell enrichment in future TCR sequencing studies in prostate cancer.

Limitations of our study are the small sample size, the focus on *BRCA2* and *ATM* mutations, the lack of tumor mutational burden measurements, and that no MDSCs were analyzed.

Collectively, our results underscore that the *BRCA2* mutation status shapes the immune phenotype of prostate cancer with an increase of intratumoral immune cells that may in part be immunosuppressive. Future strategies to prime prostate cancer for immune checkpoint therapy may hence not only focus on increasing the mutational burden, but also on manipulating immunosuppressive cell populations [54, 58].

## Electronic supplementary material

Below is the link to the electronic supplementary material.

Supplementary material 1 (PDF 65 kb)

## Abbreviations

CNA: Copy number alterations
FFPE: Formalin-fixed, paraffin-embedded
HPF: High-power field
HR: Homologous recombination
IHC: Immunohistochemistry
mCRPC: Metastatic castration-resistant prostate cancer
MDSC: Myeloid-derived suppressor cell
NGS: Next-generation sequencing
ORR: Objective response rate
OS: Overall survival
PARP: Poly-(ADP-ribose) polymerase
PFS: Progression-free survival
TCR: T-cell receptor
TIL: Tumor-infiltrating lymphocyte
