# Supplementary material for: The BRCA2 mutation status shapes the immune phenotype of prostate cancer
Source: Cancer Immunol Immunother. 2019 Sep 23;68(10):1621–33. doi: 10.1007/s00262-019-02393-x (PMC6805809; doi:10.1007/s00262-019-02393-x)
Supplement: Supplementary file 1 — Supplementary material 1 (PDF 65 kb) [file 262_2019_2393_MOESM1_ESM.pdf]

Supplementary Table 1. Overview of *BRCA2* mutations.

Patient	*BRCA2* Mutation	dbSNP	Deleterious Mutation	Germline Mutation
1	p.Gln2960*	rs80359140	+	n.t.
2	p.His2093fs*7	n/a	+	n.t.
3	p.Val1283fs*2	rs80359405	+	n.t.
4	p.Ser1951fs*11	n/a	+	n.t.
5	p.Ser1722fs*4	rs80359490	+	n.t.
6	p.Ile1247fs*12	rs886040494	+	+
7	p.Phe1182fs*1	rs80359388	+	n.t.
8	p.Pro2991_Thr3033del	n/a	+	n.t.

n.t., not tested

Supplementary Table 2. Patient characteristics.

Group	*BRCA1/2* wildtype (n=8)	*BRCA2* mutated (n=8)	p-value
BRCA1 mutations	0 (0.0%)	0 (0.0%)	
BRCA2 mutations			
Hereditary	0 (0.0%)	1 (12.5%)	
Somatic	0 (0.0%)	7 (87.5%)	
Age at Diagnosis, years, mean (SD)	62.05 (7.83)	66.59 (7.07)	0.24[a]
Initial PSA (µg/l), median (range)	23.35 (11.6-131.0)	21.00 (5.6-362.0)	0.60[a]
Initial pT stage			
pT3	8 (100.0%)	7 (87.5%)	1.00[b]
pT4	0 (0.0%)	1 (12.5%)	
Initial pN stage			
pN0	1 (12.5%)	2 (25.0%)	1.00[b]
pN1	7 (87.5%)	6 (75.0%)	
Initial c/pM stage			
c/pM0	4 (50.0%)	4 (50.0%)	1.00[b]
c/pM1	4 (50.0%)	4 (50.0%)	
Gleason-Score			
7-8	2 (25.0%)	3 (37.5%)	1.00[b]
9-10	6 (75.0%)	5 (62.5%)	
Progression free survival under ADT, months, mean (SD)	34.91 (27.11)	21.96 (22.61)	0.32[a]
Overall survival, months, mean (SD)	58.48 (30.58)	43.33 (23.35)	0.28[a]
Cancer spec. death			
Yes	7 (87.5%)	6 (75.0%)	1.00[b]

[a]Student's t-test (independent, two-sided)
[b]Fisher's exact test

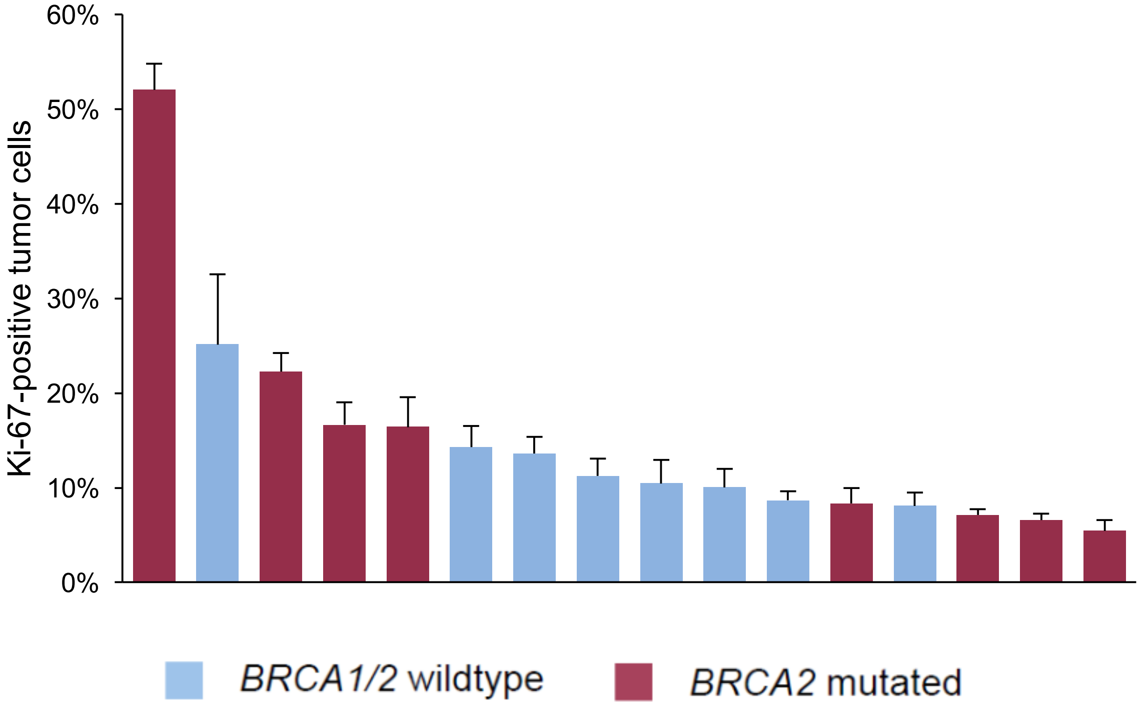

Supplementary Figure 1. Waterfall plot of the percentage of Ki-67-positive tumor cells in *BRCA1/2* wildtype (blue) and *BRCA2* mutated (red) prostate cancers.
